# Assessment of the necessity of uterine artery embolization during suction and curettage for caesarean scar pregnancy: a prospective cohort study

**DOI:** 10.1186/s12884-020-03062-z

**Published:** 2020-06-29

**Authors:** Jie Ou, Ping Peng, Chunying Li, Lirong Teng, Xinyan Liu

**Affiliations:** Department of Obstetrics and Gynecology, Peking Union Medical College Hospital, Peking Union Medical College, Chinese Academy of Medical Sciences, No. 1 ShuaiFu Road, Dongcheng District, Beijing, People’s Republic of China

**Keywords:** Caesarean scar pregnancy, Uterine artery embolism, Suction and curettage, Assessment, Fertility preservation

## Abstract

**Background:**

Uterine artery embolization (UAE) followed by suction and curettage is a common conservative treatment for caesarean scar pregnancy (CSP), but the advantages of suction and curettage alone are underestimated due to the lack of standards for selecting appropriate cases for which this approach would be applicable. We sought to identify indicators with which to assess the need for UAE during suction and curettage.

**Methods:**

The prospective cohort consisted of 105 women diagnosed with CSP in Peking Union Medical College Hospital between January 2016 and September 2018 who were followed up until 60 days after surgery. The main outcome was the therapy used, and secondary outcomes included recovery, bleeding, surgery time, length of hospital stay, and total cost.

**Results:**

We found that β-human chorionic gonadotropin (β-hCG) levels were significantly lower (*P* < 0.05), foetal cardiac activity was significantly lower (*P* < 0.05), the myometrial layer was significantly thicker (*P* < 0.05), expenditures were lower and lengths of hospital stay were shorter in patients who received suction and curettage alone (the non-UAE group) than in those who received UAE followed by suction and curettage (the UAE+ group). In addition, for CSP patients, UAE might be less necessary when the myometrial thickness is ≥2 mm and the gestational sacmeasures ≤5 cm, and suction and curettage alone may be safer for these patients.

**Conclusion:**

Suction and curettage alone is a more suitable option than UAE followed by suction and curettage because the former carries a lower cost, shorter length of hospital stay, and lower risk of adverse events. Regarding risk factors, patients with a lower uterine segment thickness ≥ 2 mm and a gestational mass diameter ≤ 5 cm have an increased probability of being successfully treated with suction and curettage alone.

## Background

Caesarean scar pregnancy (CSP) occurs when a gestational sac is implanted within the scar of a previous caesarean section [1]. Larsen and Solomon reported the first case of CSP in 1978 [2]; for most of the time since then, CSP was considered a rare disease. However, the incidence of CSP has gradually increased worldwide and now ranges from 1/2226 to 1/1899 of all pregnancies [3, 4]. Half of the reported CSP cases were published within 2015, occurring primarily in China [5]. This situation may be attributable to the high and growing frequency of caesarean section (two to four million per year) and the increasing sensitivity of ultrasound, which has improved both the timeliness and the accuracy of diagnosis [6, 7]. Coincident with the new two-child policy, concerns related to new-onset CSPs have increased in China. Similarly, other countries with high rates of caesarean delivery are likely to encounter the same problem in the near future.

CSP is a potentially high-risk complication associated with adverse events such as uterine rupture, massive haemorrhage, hysterectomy, and maternal mortality [8]. Thus, termination of pregnancy is the mainstay treatment for CSP. Although a variety of therapeutic strategies are currently applied to terminate CSPs (including medical treatments, such as systemic and/or local methotrexate (MTX); surgical treatments, such as curettage and suction, transvaginal resection of CSP, hysteroscopic surgery, laparoscopic surgery, and uterine artery embolization (UAE); and combinations of these methods [1, 9]), and these therapies can be successful individually or in combination, there is no optimal therapeutic approach, as many of the recommended strategies remain questionable due their drawbacks [10]. For example, MTX therapy is limited by toxicity to the liver, kidney, and circulatory system; a risk of vaginal bleeding; the need for a long hospital stay and close monitoring during the therapeutic timeframe; and a considerable probability of needing to be combined with supplemental treatments [11]. Furthermore, suction and curettage, whether performed with or without UAE, has been reported as a first-line treatment for CSP [4, 12].

Ultrasound-guided suction and curettage is a simple and convenient method, but previous studies have linked it to severe complications, including massive haemorrhage, uterine rupture and bladder injuries. Suction and curettage after UAE has become increasingly accepted by clinics as a conservative treatment method for CSP [13]. However, the side effects of UAE are also a source of increasing concern, as they include postoperative fever, abdominal pain, a risk of ectopic embolism, and damage to ovarian function, all of which increase the medical burden associated with this procedure; a recent study indicated a significant increase in placenta accreta spectrum (PAS) in pregnancies following UAE, and the authors suggested that previous UAE was a significant risk factor for PAS, which results in high maternal morbidity and mortality rates [14]. Over the past few years, as our experience with treating CSP has grown, we have found that many patients can be successfully treated by suction and curettage alone.

However, universal treatment guidelines for determining which patients should be treated by suction and curettage alone and knowledge of which indicators should be evaluated to assess the possible need for UAE are lacking. Based on our clinical experience and previous studies [4, 12], we hypothesized that myometrial thickness and β-human chorionic gonadotropin (β-hCG) levels may be important components in this assessment. The purpose of this study was to identify indicators that can help in assessing the need for UAE when performing suction and curettage.

## Methods

The diagnosis of CSP is based on a history of caesarean delivery, clinical manifestations, a physical examination, and the serum β-hCG level. These criteria are coupled with transvaginal ultrasonography (TVUS) criteria, as described by Timor-Tritsch [15] et al., which include (1) an empty uterine cavity and cervical canal; (2) a gestational sac located anteriorly at the level equivalent to the prior lower uterine segment of the caesarean section scar; (3) evidence of functional trophoblastic/placental circulation on Doppler scans; and (4) a negative sliding organ sign, defined as the inability to displace the gestational sac from its position at the level of the internal os.

In our hospital, ultrasound examinations were conducted by several specific experienced radiologic-gynaecologic specialists to ensure precise descriptions, including the largest dimensions of the gestational sac in terms of its length, width, and height; the thickness of the lower uterine segment’s weakest myometrial layer [16]; the vascular pattern in the scar (rich or not rich); the CSP type (type 1: in the niche, type 2: on top of the scar [17]); and foetal cardiac activity.

This prospective cohort study began by enrolling patients who received an initial diagnosis of CSP at the Peking Union Medical College Hospital between January 2016 and September 2018. The inclusion criteria were as follows: a) an initial diagnosis of CSP in our hospital; b) unwillingness to undergo MTX therapy, or indications against it; c) a gestational sac no larger than 5 cm; and d) myometrial thickness less than 1 cm. (The lower uterine segment thickness is approximately 1 cm when the uterus is extended and the lower uterine segment has formed in the third trimester, and we believe that when the lower uterine segment thickness is over 1 cm, there is a relatively low risk of terminating pregnancy in the first trimester; therefore, we chose to discuss the treatments of patients with a lower segment thickness of less than 1 cm [18].) The exclusion criteria were a) demand for caesarean scar repair at the same time, b) previous treatment in another hospital, c) a gestational sac larger than 5 cm, and d) unwillingness or inability to be followed up.

**Fig. 1:**
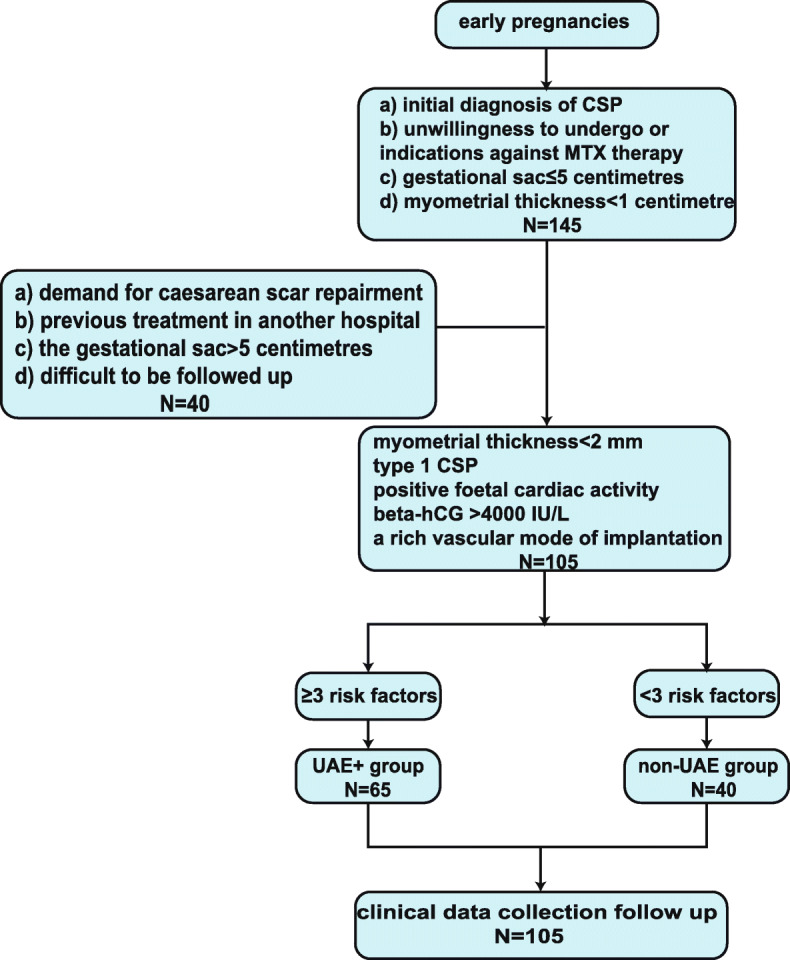
Flow chart of the study

**Fig. 2:**
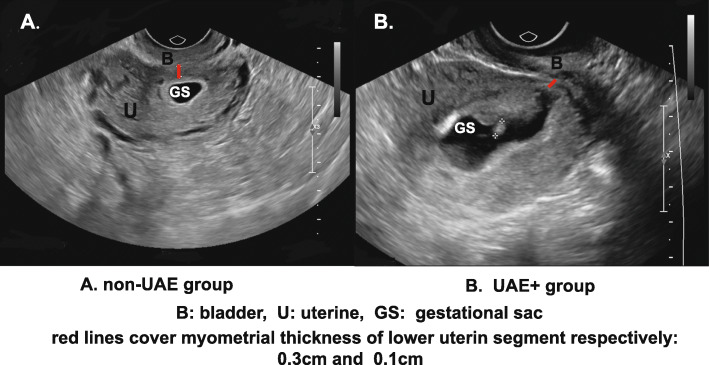
Representative ultrasonographic images from the two groups

During the study, we conducted specific treatment in the cohort after a discussion with our expert team. In the UAE+ group, the patients underwent suction and curettage following a UAE operation and had three or more of the following risk factors: myometrial thickness < 2 mm, type 1 CSP, positive foetal cardiac activity, beta-hCG > 4000 IU/L, and a rich vascular mode of implantation. All of the other patients underwent suction and curettage alone and were included in the non-UAE group. Data were collected through medical records, and the surgeries were conducted by the same group of surgeons. A flow chart of the study is shown in Fig. 1 below. Representative ultrasonographic images from the non-UAE and UAE+ groups are shown in Fig. 2.

During the study, serum β-hCG levels and venous blood haemoglobin levels were determined on the day before treatment (G0) or the next day at 6 am after curettage (G′), respectively, and then, β-hCG levels were examined weekly (Gn, where *n* = the number of weeks after surgery, starting from week 1) until recovery to normal. Blood loss was estimated by the blood volume in a vacuum jar, the area of blood on sterile gauze, and changes in haemoglobin before and after surgery. The degree of decline in β-hCG was calculated by the formula (G0-G′)/G0. Patients were also followed up in the outpatient department 2 weeks after treatment, when they underwent TVUS.

Recovery included the categories of complete cure and incomplete cure. A complete cure was defined as a complete recovery with no adjuvant treatments or severe adverse events. Complete recovery meant that β-hCG levels had decreased to normal or that the mass in the uterus had disappeared within 60 days after treatment. Adjuvant treatment included medication such as systematic/local MTX and repeat curettage or other surgical therapy. Severe adverse events included massive haemorrhage (> 500 mL) and/or hysterectomy.

UAE was performed via the right femoral artery using the Seldinger technique. A 6A5F-Yashiro catheter (Terumo, Tokyo, Japan) was advanced into the uterine arteries on both sides. Digital subtraction arteriography (AXIOM Artis FA; Siemens AG, Munich, Germany) was then performed to confirm that the catheters were correctly inserted. Subsequently, the uterine arteries were embolized with absorbable compressed sponges or particles (0.5–1.0 mm) made of gelatine. The surgeries were performed 24–72 h after UAE [19].

This study was approved by our hospital ethics committee (approval number JZ1759). Written informed consent was obtained from all patients for the use of their data in future studies. Statistical analyses were performed using SPSS version 19.0 (IBM, Armonk, NY, USA). Quantitative data are presented as the mean ± SD, frequency and percentage; between-group differences were assessed by Student’s t-test for continuous variables such as age, gestational age, and myometrial thickness or by the chi-squared test for categorical variables such as the presence of foetal cardiac activity. The influence of each indicator was assessed by binary logistic regression analysis. *P*-values < 0.05 were considered to indicate a statistically significant difference.

## Results

In our study, 145 patients were diagnosed with CSP. Among these patients, 105 women were enrolled in the cohort, and 40 were excluded because of the thickness of the myometrial layer (21 patients), the diameter of the gestational sac (14 patients) or unwillingness to undergo MTX therapy (5 patients). The patients in this cohort had an average age of 33.7 ± 0.44 years (range, 25 to 44 years) and a mean gravidity of 3.7 ± 0.14 (range, 2 to 9). Among the patients, 74 (70.5%), 30 (28.6%), and 1 (0.9%) had had 1, 2, and 3 previous caesarean deliveries, respectively. The gestational age ranged from 4 to 15 weeks and averaged 7.4 ± 0.15 weeks. Additionally, the mean thickness of the myometrial layer at the caesarean scar was 0.25 ± 0.013 cm (range, 0.01 to 0.66 cm). The median β-hCG level was 82,977 ± 8050 IU/L (range, 291 to 404,700 IU/L), and the mean diameter of the gestational sac was 2.52 ± 0.10 cm (range, 1 to 5 cm).

Ultimately, the 65 patients in the UAE+ group successfully underwent UAE and curettage, and the 40 patients in the non-UAE group underwent curettage alone under the premise of preparation for UAE and other methods in case of massive bleeding; thus, the ratio of non-UAE to UAE+ patients was approximately 2:3. Age, gravidity, parity, gestational age, number of previous uterine operations, number of previous caesarean deliveries (CDs), and the interval between CD and CSP were not significantly different between the two groups. On TVUS, the lengths of the gestational sacs were 2.41 ± 1.02 and 2.59 ± 0.98 cm in the non-UAE and UAE+ groups, respectively. In all, foetal cardiac activity was evident in 34 (52.3%) gestational sacs in the UAE+ group and 13 (32.5%) in the non-UAE group. In the UAE+ group, there were 13 (20.0%) cases of type 1 CSP and 52 (80.0%) cases of type 2 CSP, while in the non-UAE group, there were 3 (7.5%) cases of type 1 CSP and 37 (92.5%) cases of type 2 CSP. Additionally, 55 (84.6%) and 31 (77.5%) of the patients in the UAE+ group and non-UAE group, respectively, had a rich vascular pattern in the scar. In conclusion, there was no significant intergroup difference in the number of cases with a rich vascular pattern, the size of the mass in the uterus, or the type of mass. However, the non-UAE group had significantly lower β-hCG levels (*P* < 0.05), a significantly lower rate of foetal cardiac activity (*P* < 0.05), and a significantly thicker myometrial layer than the UAE+ group (*P* < 0.05). The clinical characteristics of the two groups are summarized in Table 1.

**Table 1:** Patient clinical data in the two groups

	Non-UAE (*N* = 40)	UAE+ (*N* = 65)	*P* value
Age (years)	33.4 ± 4.8	34.0 ± 4.4	0.535
Gestational age (weeks)	7.21 ± 1.62	7.45 ± 1.47	0.439
Gravidity	3.63 ± 1.33	3.77 ± 1.49	0.687
Parity	1.38 ± 0.49	1.31 ± 0.49	0.404
Previous UOs	2.33 ± 1.21	2.72 ± 1.53	0.142
Previous CDs	1.35 ± 0.48	1.28 ± 0.49	0.454
Time interval (years)	3.48 ± 2.15	4.06 ± 2.42	0.199
Myometrial thickness (cm)^a^	0.349 ± 0.122	0.182 ± 0.087	0.001
RVP	31	55	0.358
Mass size (cm)	2.41 ± 1.02	2.59 ± 0.98	0.373
FCA^a^	13	34	0.047
Type 2 CSP: on top of the scar	37	52	0.083
β-hCG^a^ (IU/L)	58,956.5 ± 77,262	97,990.5 ± 84,802	0.014

Abbreviations: *UO* uterine operation, *UAE* Uterine artery embolization, *CD* caesarean delivery, *RVP* rich vascular pattern, *FCA* foetal cardiac activity, *β-hCG* β-human chorionic gonadotropin; time interval, time elapsed between CD and CSP. ^a^indicates that the item was significantly different between the two groups

Finally, in the non-UAE group, no patient was lost to follow-up, and 36 (90%) of the patients recovered successfully after a single procedure. Of the remaining women, 2 (5%) received systematic MTX at a dose of 75 mg/m^2^, and 2 (5%) underwent curettage again due to a persistent mass in the uterus and an unsatisfactory decline in β-hCG levels 2 weeks after the first surgery; all four of those patients ultimately achieved a successful recovery. In the UAE+ group, 64 (98%) of the patients recovered successfully after a single procedure, and only 1 (2%) patient underwent repeat suction and curettage. There were no cases of uterine perforation during ultrasound-guided dilation and curettage (D&C). Five patients complained of abdominal pain, which subsided after intravenous analgesia; 10 patients suffered from fever of unknown aetiology, with temperatures ranging from 37.7 to 38.1 °C, and recovered within 72 h by physical cooling without the use of antibiotics. There were no cases of embolism, uterine infection with amenorrhea, or premature ovarian failure during follow-up.

Overall, there was no significant difference in recovery between the groups, and none of the women in either group experienced severe adverse events, such as hysterectomy or massive bleeding.

The durations of surgery in the non-UAE and UAE+ groups were 29.0 ± 3.0 min and 29.8 ± 1.3 min, respectively, while the bleeding volumes were 22.9 ± 16.3 ml and 26.3 ± 41.5 ml, respectively; these differences were not significant. The lengths of hospitalization were 2.53 ± 1.22 days in the non-UAE group and 4.48 ± 1.95 days in the UAE+ group, and this difference was significant (*P* < 0.05). Similar results were found for expenditures: in the UAE+ group, the cost was 18,723 ± 2671 CNY (Chinese yuan), which was significantly higher than the cost in the non-UAE group (3438 ± 2970 CNY) (*P* < 0.05). The outcomes obtained in the two groups are shown in Table 2.

**Table 2:** Outcomes in the two groups

	Non-UAE (*N* = 40)	UAE+ (*N* = 65)	*P* value
hCG decline (%)^a^	70.3 ± 17.8	78.1 ± 17.8	0.033
Successful case	36 (90%)	64 (98%)	0.132
Unsuccessful case	4	1	
MTX	2	1	
Repeat D&C	2	1	
Severe adverse event	0	0	
Bleeding (ml)	22.9 ± 16.3	26.3 ± 41.5	0.558
Surgery time (min)	29.0 ± 3.0	29.8 ± 1.3	0.101
Inpatient time (d)^a^	2.53 ± 1.22	4.48 ± 1.95	0.000
Expenditure (CNY)^a^	3438 ± 2970	18,723 ± 2671	0.000

^a^indicates that the item was significantly different between the two groups; *CNY* Chinese yuan.

We chose a myometrial thickness of 2 mm as the cut-off point for evaluating our patients. Logistic regression analysis was used to determine the associations among the three indicators—myometrial thickness ≥ 2 mm, β-hCG ≤4000 IU/L, and foetal cardiac activity—and their degree of utility in assessing the need for UAE. These results are shown in Table 3.

**Table 3:** Logistic regression analysis results

	Non-UAE	UAE+	*P* value	OR
Myometrial thickness^a^ ≥2 mm			0.002	1.14 (1.04, 1.63)
β-hCG < 4000			0.163	–
FCA			0.074	–

Abbreviations: *FCA* foetal cardiac activity, *β-hCG* β-human chorionic gonadotropin; ^a^ indicates that the item was significantly different between the two groups

## Discussion

To date, more than 50 studies of CSP treatment have been published, although a majority of recommendations are still based on case series rather than randomized controlled trials (RCTs) [5].

The management of CSP can be surgical or pharmacological, with the latter mainly consisting of systemic or local administration of MTX. Surgical management includes D&C, UAE, hysteroscopy, and laparoscopic management to excise the gestational sac from the uterine scar [10]. Hysteroscopy/laparoscopy management is characterized by short follow-up and a rapid normalization of β-hCG; however, a skilled surgeon and a haemodynamically stable patient are essential for the procedure [20]. MTX is one of the alternative treatments for haemodynamically stable patients without pain, but the reabsorption of the gestational sac takes a long time, and there is a risk of massive haemorrhage and a need for additional treatments; these drawbacks may make patients reluctant to undergo this method [21].

A recent review by Timor-Tritsch and Monteagudo reported that UAE combined with D&C had increased rates of less severe complications— specifically, fever and mild pain in the abdomen or the pelvic region—for an overall complication rate of 46.9% [15], mostly related to UAE. In our study, 65 patients underwent UAE and curettage, among whom 5 patients complained of abdominal pain and 10 patients suffered from fever of unknown aetiology, with a total complication rate of 20%.

Suction and curettage is an appropriate treatment due to its low cost, practicality, effectiveness, low rate of side effects, and minimal influence on future fertility. However, its application is still controversial in the literature. Polat et al. [10] reported massive haemorrhage associated with suction and curettage in one patient. Another report indicated that primary suction and curettage had a complication rate of 61.9%, including uterine perforation and massive bleeding [7]. In contrast, Weilin and Li [22] indicated suction and curettage as a feasible and effective method for lower-risk endogenous CSP patients. The reason for such major discord between different reports is that they did not assess the same factors to determine which patients could be treated with suction and curettage alone.

In our study, we aim to identify indicators with which to assess the need for UAE during suction and curettage or D&C in CSP patients so that we can minimize the side effects of UAE and maximize the advantages of curettage and suction.

Both groups achieved satisfactory outcomes (complete cure in over 90% of cases and no severe complications in any case), which were not significantly different between the UAE+ group and the non-UAE group, indicating that the treatments we conducted in this cohort in our hospital were appropriate and effective. Specifically, the patients in the non-UAE group had significantly shorter lengths of stay (*P* < 0.05), lower costs (*P* < 0.05), and slower declines in β-hCG levels (*P* < 0.05) than the UAE+ patients. Furthermore, women in the non-UAE group were characterized by significantly thicker myometrial thickness (*P* < 0.05), lower β-hCG levels (*P* < 0.05), and less foetal cardiac activity (*P* < 0.05) than those in the UAE+ group. In addition, our study suggests that when the myometrial thickness is ≥2 mm, UAE is not likely to be necessary, and it is safer to perform suction and curettage alone.

Sheng Wang [4] reported a retrospective review of 240 patients with CSP who were grouped according to two classes of management options: ultrasound-guided suction curettage alone and in combination with other therapeutic options. The review indicated that in CSP patients with a lower uterine segment myometrium thickness of more than 2 mm, ultrasound-guided suction and curettage appeared to be a reliable treatment option, Thus, dose studies by Polat I. and Shao M [4, 12, 23]. conclude that surgical approaches in the treatment of CSPs using 2 mm boundaries may yield an optimal clinical outcome.

Myometrial thickness indicates the proportions of smooth muscle and fibrous tissue, which reflect the ability of the caesarean scar to contract and the ability of the tissue to stop bleeding when the pregnancy-related tissue is removed. According to this hypothesis, it is reasonable to propose that myometrial thickness is an important indicator of the necessity for UAE. While the hCG level and foetal cardiac activity reflect the activity of trophoblast cells and foetal tissues, respectively, these are not directly associated with the degree of trophoblast cell invasion into the uterine myometrium.

However, one limitation of our study is its nature as a cohort study. Specifically, this study enrolled only patients with a myometrial thickness over 0.1 cm and a gestational sac with a diameter less than 5 cm, and the conclusions of this study, therefore, cannot be extended to every patient diagnosed with CSP. It might also be ethically inappropriate to conduct a random blinded clinical trial among patients with CSP because UAE is an invasive operation that is very expensive. In addition, because the decision to perform curettage or curettage plus UAE was not random but was instead made by experts, there might have been selection bias in this study.

## Conclusions

Early diagnosis and appropriate therapy contribute to the successful management of CSP. Suction and curettage alone is a suitable option because of its low cost, short hospital stay, low risk of adverse events and low influence on fertility [24]. Patients with a lower uterine segment thickness of more than 2 mm and a gestational mass with a diameter less than 5 cm can be successfully treated with suction and curettage alone, but physicians must inform patients of the risk of potential complications and be prepared for emergency salvage procedures.

## Data Availability

The datasets used and/or analysed during the current study are available from the corresponding author on reasonable request.

## Abbreviations

CD: Caesarean delivery
CSP: Caesarean scar pregnancy
CNY: Chinese yuan
D&C: Dilation and curettage
FCA: Foetal cardiac activity
MTX: Methotrexate
RVP: Rich vascular pattern
TVUS: Transvaginal ultrasonography
UAE: Uterine artery embolization
UO: Uterine operation
β-hCG: β-human chorionic gonadotropin
